# The Morrell Mackenzie Controversy

**Published:** 1893-06-17

**Authors:** 


### The Hospital

An Institutional Journal of

Science, Medicine, Nursing, and Philanthropy.

*For Hospitals, Asylums, Infirmaries, Dispensaries, Medical Practitioners, Students, Nurses, and the Charitable Public.*

Vol. XIV.—No. 351.] June 17, 1893.

### The Morell Mackenzie Controversy.

Persons who value the honour of the medical profession have been made somewhat unhappy during the last week or two by the letters of the Mackenzie family and of the Rev. H. R. Haweis, which have been published, and by the newspaper paragraphs which have been written thereupon. We do not propose to open up old controversies, but to make use of the occasion for the consideration of the general attitude of the leaders of the medical proprofession in dealing with circumstances which lie a little off the usual lines of professional life.

The profession of medicine has now attained to a position in the public estimation which demands of its leaders not only soundness of judgment but breadth; and not only breadth and soundness of judgment, but steadiness and consistency in action. The leaders of medicine in the past have often shown an entire lack of judicial capacity, and, by consequence, an impulsiveness of action which has made the great world smile, but has produced in the smaller world of medicine a feeling of just anger rather than of amusement. Take this very case of Sir Morell Mackenzie as an illustration. Sir Morell thought it right, in the exercise of his discretion, to pursue a course with regard to certain German medical men which many leading members of the medical profession disapproved; and, as we think, rightly disapproved. But individual disapproval is one thing; the proceeding to official and public acts which may permanently damage a man's reputation and injure his money-earning power is a question of another character altogether. Sir Morell Mackenzie, if we remember rightly, was requested to appear before the authorities of the Royal College of Physicians. Such a summons ought to have been the very thing he most wished. He ought to have felt a degree of confidence in the judgment and right feeling of those authorities which would have caused him to desire above all things to lay his case before them in the full assurance that they would vindicate him if he could be vindicated; and that, in any case, they would do him absolute justice, and would temper their justice with the charity of professional brotherhood, and with the worldly wisdom which comes of liberal culture, and of familiarity with public affairs.

But, whether rightly or not, it seems certain that Sir Morell Mackenzie did not place in the judgment, charity, and worldly wisdom of the authorities of the College of Physicians such confidence as he ought to have been able to place. There seems to be little doubt that he felt that he was summoned before a tribunal which was not likely to take a broad and dispassionate view of his case; a tribunal which was resolved beforehand not to hear him and to vindicate him if possible, but by all means to submit him to the humiliation and public injury of an expression of disapproval. Rather than submit to that, Sir Morell Mackenzie severed his connection with the Royal College of Physicians.

Now, how far Sir Morell may or may not have been right in these feelings which, without special knowledge, we have ascribed to him, it is impossible to say. But this we can affirm, that there is a widespread feeling abroad in the medical profession that the Royal College of Physicians is not a liberal body, is not a judicial body, is not a body of wide experience in affairs, and is not in true and brotherly sympathy with the medical profession as a whole.

That is a state of things which should not now exist, and which ought never to have existed. But at any rate, it is a condition of things which the whole profession is interested in putting an end to. The medical profession as a whole is greater than the College of Physicians, since the whole is and must be greater than its part. The College of Physicians happens to represent the head of the medical body; but exactly because it does represent the head must the body be critical and watchful, in order that the head may bring honour and not discredit to the organism.

In the purely medical capacity of the heads and fellows of the Royal College of Physicians, the profession as a whole has a very great amount of confidence. But in the judicial capacity of the College, and in its knowledge of general affairs, and its fitness for dealing with them, there is by no means the same happy trust. But the profession is most urgently desirous that there should be that trust. The thirty thousand men of culture who constitute the profession feel themselves to be somewhat at the mercy of circumstances in this matter. They have no voice in the appointment of the heads of the College of Physicians; they have no voice in the selection of men for the honour of its fellowship. The College is, for all practical purposes, a private corporation, with its own private rules, customs, and traditions. Moreover, it can only be disinterested with the greatest possible difficulty. The interests of its fellows are more or less antagonistic to those of the body of the profession. The fellows have everything to gain from a pecuniary standpoint by exalting their own importance and minimising the professional worth of those who are outside. Besides, there is no rival of equal status with the College; no Academy of Medicine which might hold its excessive pretensions in reasonable check.

This, then, is the position which the Royal College of Physicians has to face: that the profession desires to give it both respect and confidence, but sometimes finds it exceedingly difficult to do so. The weakness of the College, as we have said, lies in the incompetency of its judicial faculty. It is impulsive; it is not statesmanlike; it rushes forward with unreasoning haste; and then has to retrace its steps without dignity. To recur to the example of Sir Morell Mackenzie, it was clearly a case from which personal feeling should have been altogether eliminated; but as clearly was it a case in which personal feeling rose to flood-tide, and threatened to overflow the banks of reason, justice, and common-sense altogether. But the leaders of the College should have been sensitively alive to the electrical atmosphere in which they were living, and should have insisted either upon deferring the consideration of Sir Morell Mackenzie's case for at least a year, or of calling in the aid of competent legal advisers, who would have taken evidence competently and dispassionately, and given their decision as judges and experienced men of the world, and not as interested partisans.

Perhaps those who know the Rev. H. R. Haweis well do not regard him as the most judicious or even as the least egotistical of human beings; and his pronouncements on the College-Mackenzie case need not therefore make the representatives of the College permanently melancholy. Nevertheless, as representing the head of the profession, in England at any rate, the College ought to be like Cæsar's wife, absolutely above suspicion. It is extremely careful of the honour of its individual members; but it appears to have altogether too little appreciation of the far more important circumstance of its corporate honour. It ought not to have been possible for even the Rev. H. R. Haweis to bring a railing accusation against the College, and indirectly through it against the medical profession as a whole. We must hope that the recollection of the Morell Mackenzie incident will long remain in the minds of the heads of the College, and will be productive of a judicial and worldly-wise attitude of mind which will permanently conserve the honour of the College itself and of the whole medical profession.

